# Supplementary material for: Full-Length Transcriptome Sequencing Reveals Treg-Specific Isoform Expression upon Activation
Source: Int J Mol Sci. 2025 Jun 30;26(13):6302. doi: 10.3390/ijms26136302 (PMC12249553; doi:10.3390/ijms26136302)
Supplement: Supplementary file 1 [file ijms-26-06302-s001.zip › Supplementary Figures.pdf]

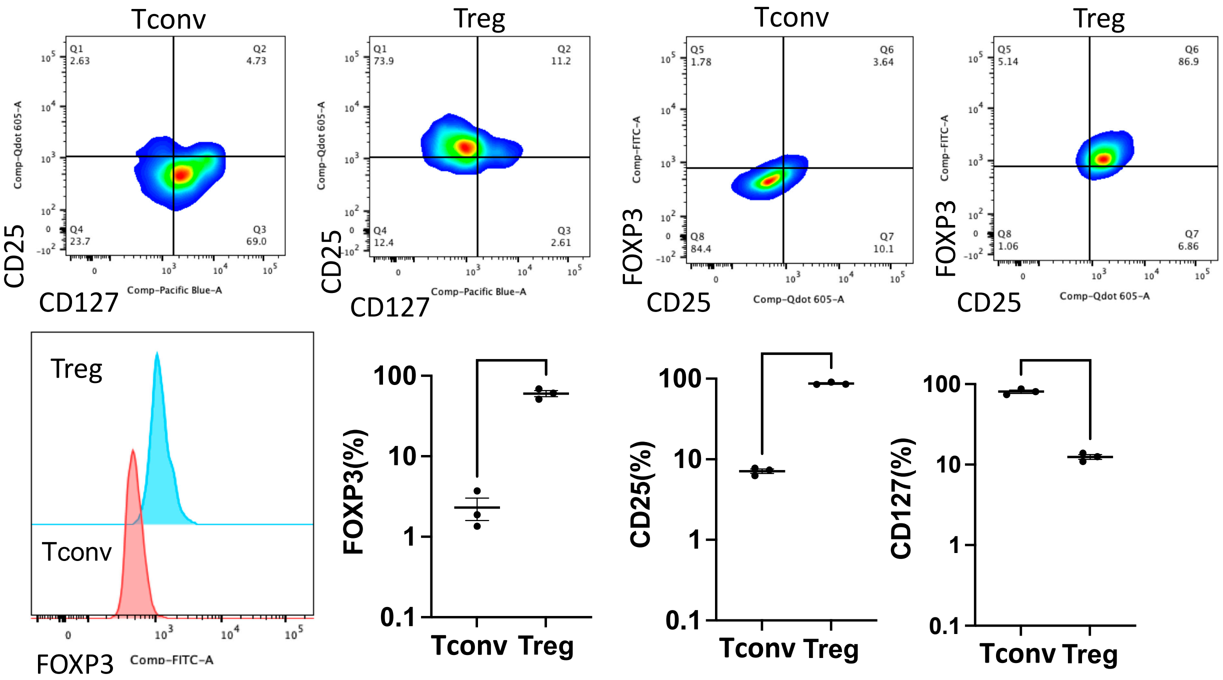

Supplementary Figure S1 Comparison of the phenotype for Tregs and Tconvs among different donors. Tregs (CD4^{+}CD25^{+}CD127^{low}) and Tconvs (CD4^{+}CD25^{-}) were identified and isolated by fluorescence activated single cell sorting (FACS). The phenotype of Tregs and Tconvs were comparable among different donors (n=3, Mean±SEM).

Direction	GSEA analysis: treg vs teff	NES	Genes	adj.Pval
Down	GSE11057 NAIVE VS MEMORY CD4 TCELL UP	-2.6505	154	7.2e-04
	GSE25087 TREG VS TCONV ADULT DN	-2.6297	152	7.2e-04
	GSE45739 UNSTIM VS ACD3 ACD28 STIM WT CD4 TCELL DN	-2.4749	174	7.2e-04
	GSE5542 UNTREATED VS IFNA TREATED EPITHELIAL CELLS 6H DN	-2.4399	172	7.2e-04
Up	GSE36476 CTRL VS TSST ACT 40H MEMORY CD4 TCELL YOUNG DN	2.9864	176	7.2e-04
	GSE24634 TREG VS TCONV POST DAY7 IL4 CONVERSION UP	2.9291	165	7.2e-04
	GSE36476 CTRL VS TSST ACT 72H MEMORY CD4 TCELL YOUNG DN	2.9129	177	7.2e-04
	GSE36476 CTRL VS TSST ACT 72H MEMORY CD4 TCELL OLD DN	2.8688	177	7.2e-04
	GSE15750 DAY6 VS DAY10 TRAF6KO EFF CD8 TCELL UP	2.8594	176	7.2e-04
	GSE24634 TEFF VS TCONV DAY7 IN CULTURE UP	2.8505	178	7.2e-04
	GSE36476 CTRL VS TSST ACT 40H MEMORY CD4 TCELL OLD DN	2.8259	179	7.2e-04
	GSE25087 TREG VS TCONV ADULT UP	2.7778	167	7.2e-04
	GSE15750 DAY6 VS DAY10 EFF CD8 TCELL UP	2.7516	173	7.2e-04
	GSE21063 WT VS NFATC1 KO 8H ANTI IGM STIM BCELL UP	2.7183	168	7.2e-04
	GSE30962 PRIMARY VS SECONDARY ACUTE LCMV INF CD8 TCELL UP	2.6661	171	7.2e-04
	GSE45365 HEALTHY VS MCMV INFECTION CD11B DC DN	2.664	105	7.2e-04
	GSE14415 INDUCED VS NATURAL TREG DN	2.5951	158	7.2e-04
	GSE24634 TREG VS TCONV POST DAY10 IL4 CONVERSION UP	2.5925	183	7.2e-04
	GSE39110 DAY3 VS DAY6 POST IMMUNIZATION CD8 TCELL DN	2.5866	169	7.2e-04
	GSE13547 CTRL VS ANTI IGM STIM BCELL 12H UP	2.5734	170	7.2e-04
	GSE13738 RESTING VS BYSTANDER ACTIVATED CD4 TCELL DN	2.5734	179	7.2e-04
	GSE25087 TREG VS TCONV FETUS UP	2.5551	168	7.2e-04
	GSE39556 CD8A DC VS NK CELL MOUSE 3H POST POLYIC INJ UP	2.5495	175	7.2e-04
	GSE14415 NATURAL TREG VS TCONV DN	2.5079	153	7.2e-04
	GSE24634 NAIVE CD4 TCELL VS DAY10 IL4 CONV TREG DN	2.4965	175	7.2e-04
	GSE21063 CTRL VS ANTI IGM STIM BCELL NFATC1 KO 8H DN	2.4753	174	7.2e-04
	GSE29614 CTRL VS DAY7 TIV FLU VACCINE PBMC DN	2.4672	150	7.2e-04
	GSE24634 NAIVE CD4 TCELL VS DAY7 IL4 CONV TREG DN	2.4573	175	7.2e-04
	GSE14415 INDUCED TREG VS TCONV UP	2.4491	158	7.2e-04
	GSE24634 IL4 VS CTRL TREATED NAIVE CD4 TCELL DAY7 UP	2.4381	151	7.2e-04

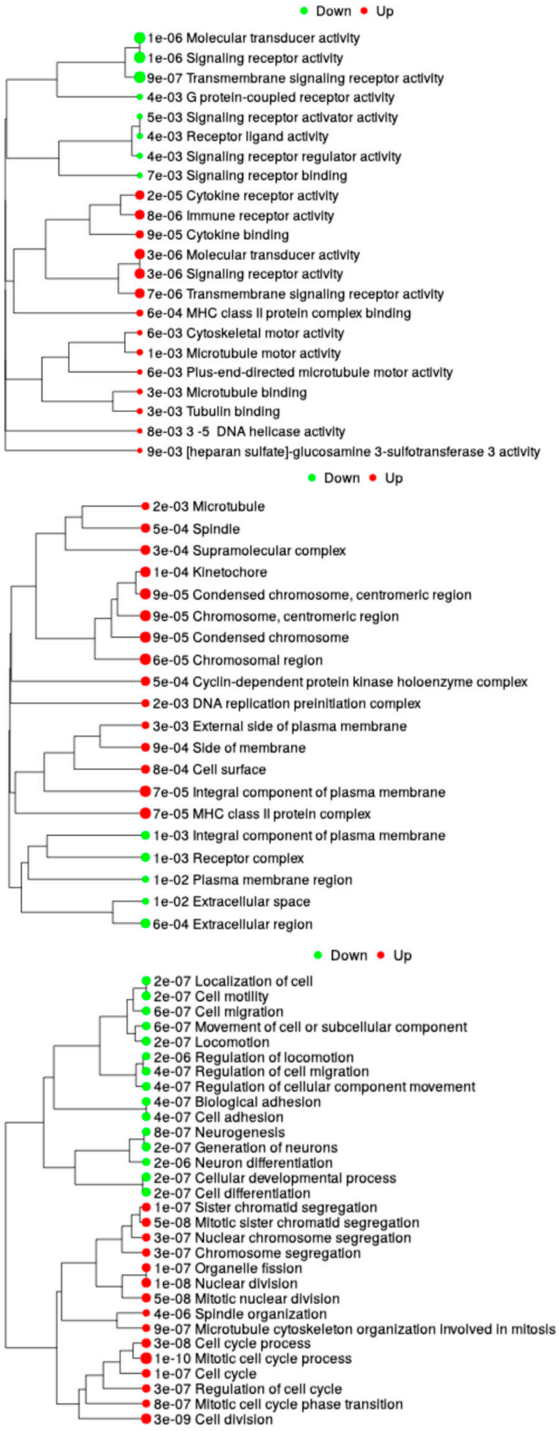

Supplementary Figure S2 RNA-seq analysis of Tregs. List of GSEA (C7: immunologic signature gene sets). Gene ontology analysis (Biological Process, Molecular Function, and Cellular Compartment).

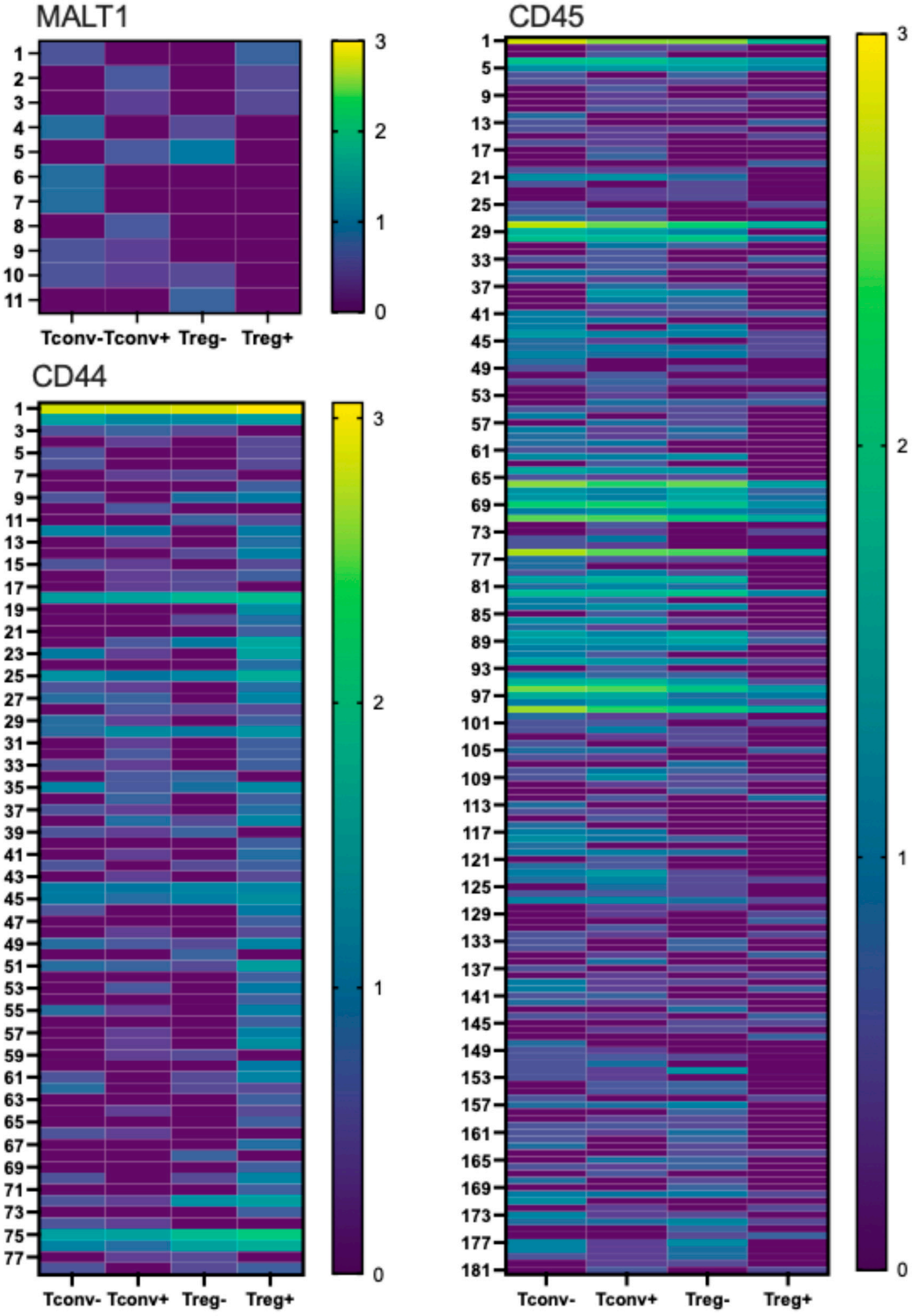

Supplementary Figure S3 T cells expressing multiple isoforms. Iso-seq analysis of representative genes (MALT1, CD44, and CD45) known to express multiple isoforms.

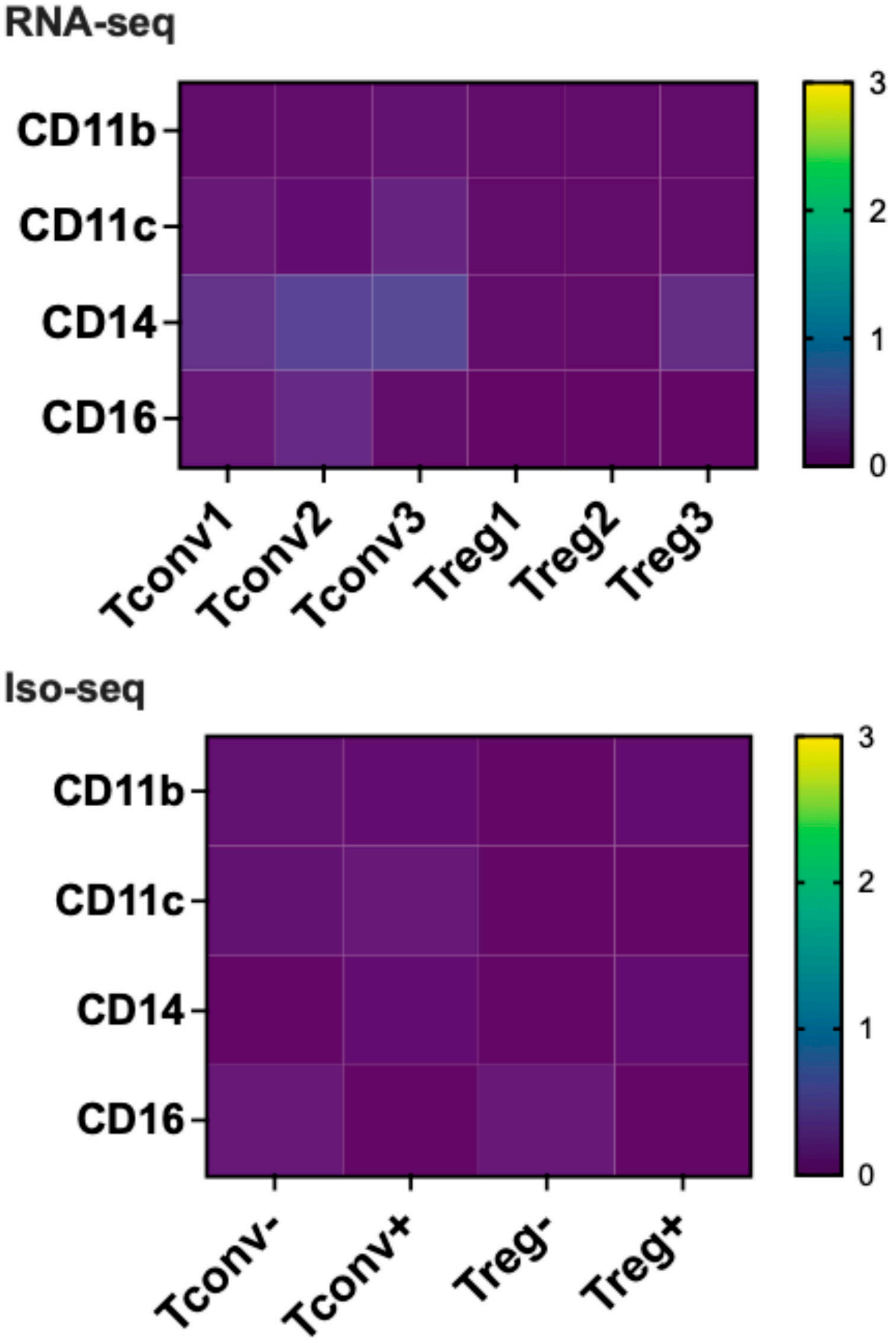

Supplementary Figure S4 RNA-seq and Iso-seq analyses did not detect antigen-presenting cells among Tregs. Bulk RNA-seq and Iso-seq showed minimal expression of representative genes of the antigen-presenting cells.

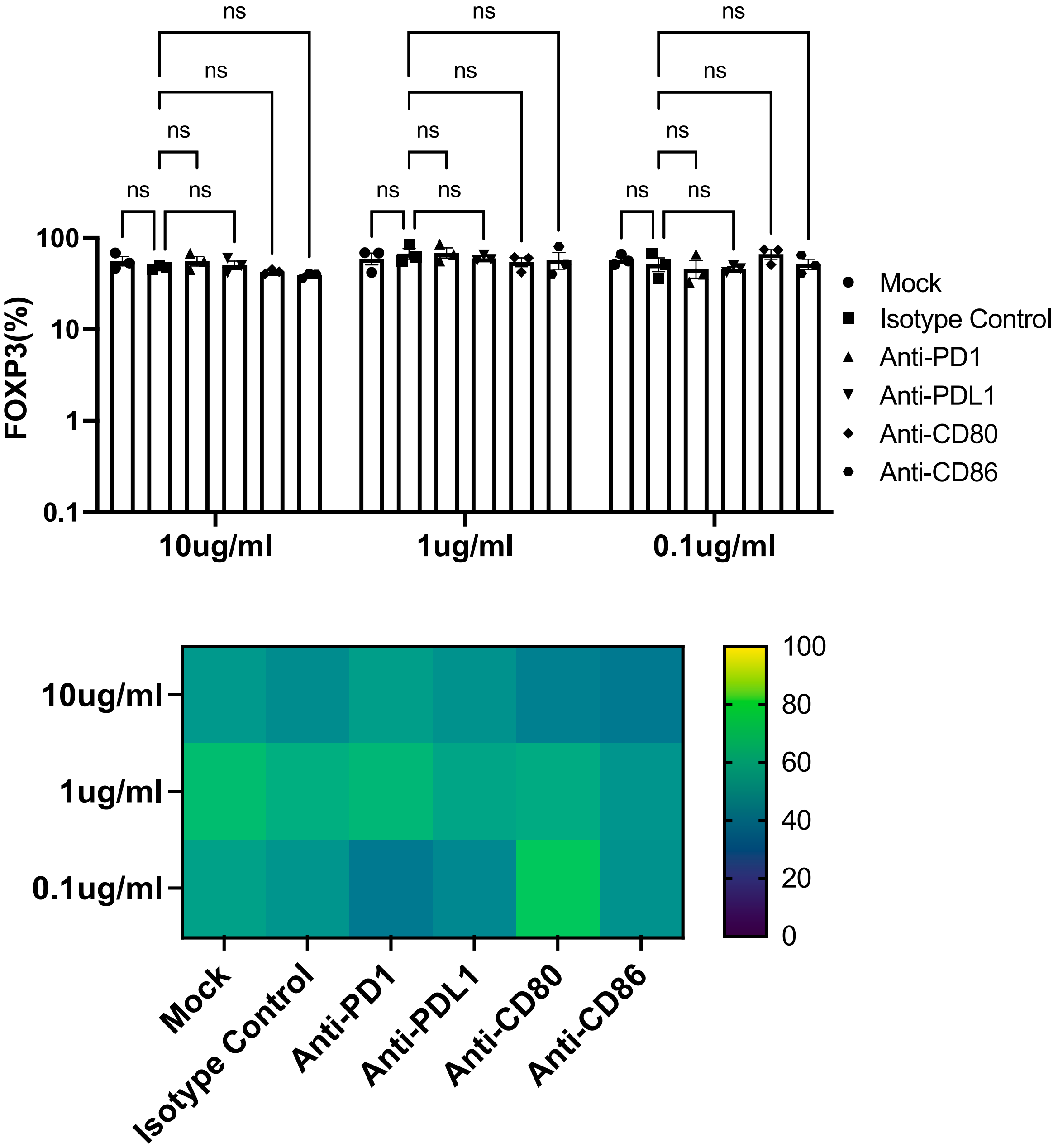

Supplementary Figure S5 Treatment of Tregs with antibodies against PD-1, PD-L1, CD80, and CD86 did not diminish FOXP3 expression. Tregs were incubated with PD-1, PD-L1, CD80, and CD86 antibodies, and FOXP3 expression was measured using fluorescence activated single cell sorting (FACS) (n=3, Mean±SEM).
